# Diffuse sulcal hyperintensity on MRI as a radiological correlate of transient neurological deficits in chronic subdural hematoma

**DOI:** 10.1186/s12883-026-04913-6

**Published:** 2026-04-23

**Authors:** Shota Yoshimura, Susumu Yamaguchi, Hikaru Nakamura, Kosuke Hirayama, Yukishige Hayashi, Takayuki Matsuo

**Affiliations:** 1 Department of Neurosurgery, Nagasaki Prefecture Shimabara Hospital, 7895, Shimokawajiri-machi, Shimabara, 855-0861 Japan; 2 Department of Neurosurgery, Nagasaki Harbor Medical Center, 6-39, Shinchi- machi, Nagasaki, 850-8555 Japan; 3 Department of Neurosurgery, Graduate School of Biomedical Sciences, Nagasaki University, 1-7-1, Sakamoto, Nagasaki, 852-8501 Japan

**Keywords:** chronic subdural hematoma, sulcal hyperintensity, transient neurological deficits, magnetic resonance imaging, fluid attenuated inversion recovery

## Abstract

**Background:**

Transient neurological deficits (TND) may present as the initial symptom of chronic subdural hematoma (CSDH). Although a relationship between sulcal hyperintensity (SHI) on FLAIR images and TND has been suggested, existing evidence is limited to case reports and small-scale cohort studies. This study aimed to investigate the association between diffuse SHI and TND in patients with CSDH.

**Methods:**

This single-center retrospective study included 127 patients with CSDH who underwent perioperative CT and MRI including FLAIR sequences, between January 2017 and March 2025. To identify factors associated with TND, univariate and multivariable logistic regression analyses were used to estimate odds ratios (ORs) with 95% confidence intervals (CIs). SHI on FLAIR was defined as marked hyperintensity along cerebral sulci adjacent to the hematoma, categorized as diffuse or focal.

**Results:**

Among the 127 patients (median age, 82 [74–86] years, 86 male) with CSDH, 22 (median age, 81 [72–85] years, 17 male) presented with diffuse SHI. Of the 22 patients with diffuse SHI, nine (40.91%) underwent TND. The presence of diffuse SHI was independently associated with TND (adjusted OR; 17.07, 95% CI; 4.71–73.23, *p* < 0.01).

**Conclusions:**

This study demonstrates a significant correlation between preoperative TND and diffuse SHI in patients with CSDH. These findings may serve as a potential biomarker for TND in patients with CSDH, providing useful information that can complement existing diagnostic methods.

**Supplementary Information:**

The online version contains supplementary material available at 10.1186/s12883-026-04913-6.

## Introduction

Chronic subdural hematoma (CSDH) is a condition predominantly observed in the elderly population, characterized by the blood collection between the inner and outer membranes [1]. Recently, randomized controlled trials on middle meningeal artery embolization as a treatment for CSDH have attracted attention [2, 3]. Increased CSDH can lead to the compression of the brain parenchyma, resulting in neurological symptoms such as headaches, nausea, motor disturbances, cognitive deficits, altered consciousness, language impairments, and seizures [4]. Transient neurological deficits (TND) may sometimes present as the initial symptom, requiring differentiation from transient ischemic attack, epilepsy, migraine, demyelinating diseases, hypertensive encephalopathy, blood glucose and electrolyte abnormalities, and psychiatric disorders [5, 6]. Therefore, magnetic resonance imaging (MRI) is often performed during the initial examination. The prevalence of TND varies significantly among reports, ranging from 1 to 24% among CSDH, depending on the definition [7]. The most frequently observed TND in clinical practice are aphasia or dysarthria, followed by unilateral weakness or unilateral sensory disturbance [7]. The pathophysiological mechanisms involved in the development of TND are diverse and remain unclear, including cortical spreading depression, local ischemia, recurrent microbleeds, mechanical compression by hematoma, and epileptic activity [8].

Sulcal hyperintensity (SHI) observed on fluid attenuated inversion recovery (FLAIR) imaging in MRI may indicate irritative alterations in the brain parenchyma or cortex, and a correlation with TND or seizures has been proposed [6, 9]. However, only a limited number of systematic studies have examined the relationship between SHI and TND, with the available reports restricted to case reports and small retrospective cohort studies [10–12]. This study aimed to investigate the relationship between diffuse SHI and TND in CSDH and perform a preliminary evaluation of its potential as a diagnostic biomarker.

## Methods

### Study design and participants

**Fig. 1:**
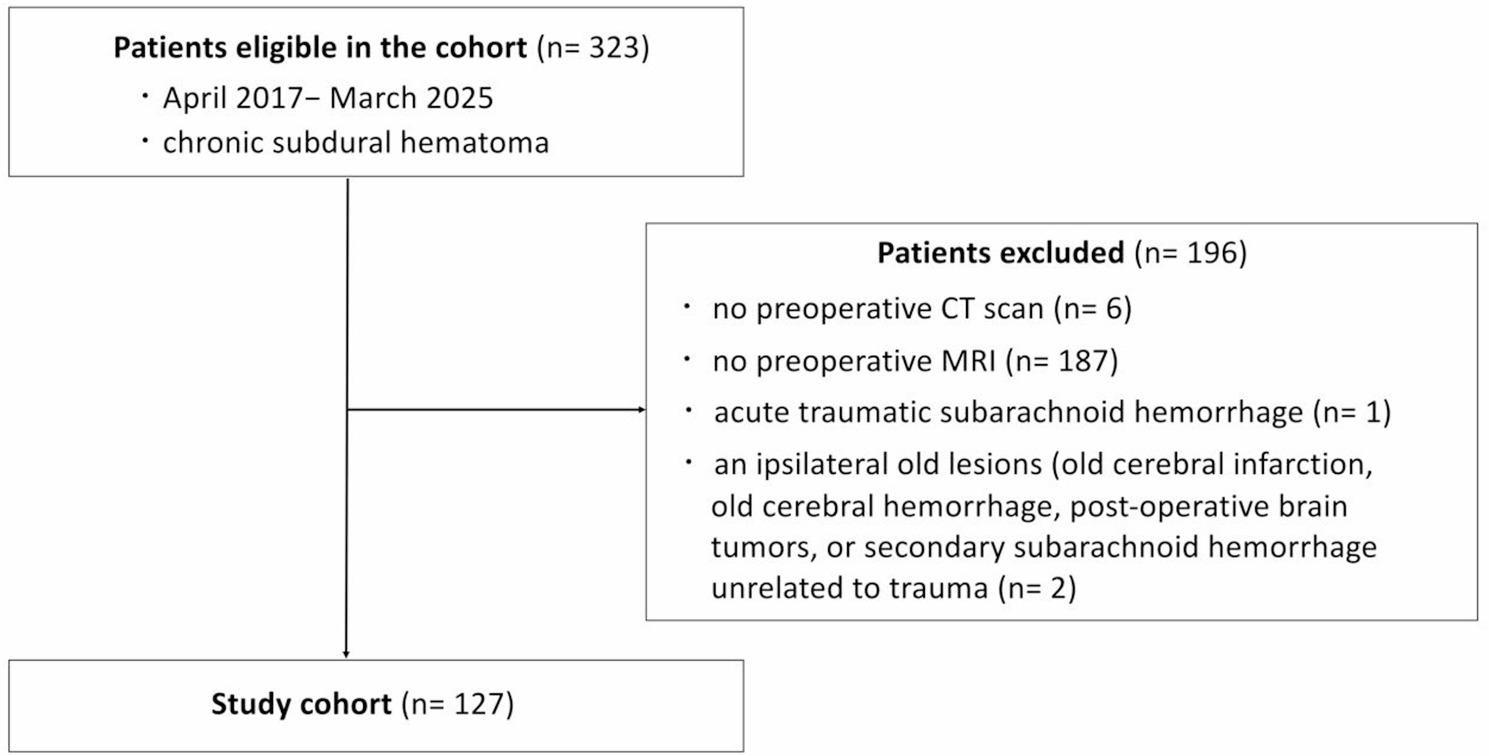
Flowchart of the study population

Among the 323 patients with CSDH who underwent surgery at our hospital between January 2017 and March 2025, we included those who underwent computed tomography (CT) and MRI preoperatively in Fig. 1. We excluded patients with acute traumatic subarachnoid hemorrhage, a history of intracranial lesions on the same side as the chronic subdural hematoma, such as old cerebral hemorrhage, old cerebral infarction, brain tumor), and secondary subarachnoid hemorrhage unrelated to by trauma. This study included 127 patients. Background factors, such as age, sex, preexisting diseases, use of antithrombotic drugs, main symptoms, CSDH recurrence, and modified Rankin Scale (mRS) score, were examined. Functional outcome was evaluated using the mRS score, which ranges from 0 (no symptoms) to 6 (death). The mRS score reflects the level of functional disability and dependence in activities of daily living and was determined by trained assessors using standardized criteria [13]. The mRS score was assessed before treatment and at discharge.

TND includes all transient signs and symptoms, excluding transient symptoms, such as headache, nausea, and vomiting. Previous reports of CSDH with TND have indicated that transient disturbances of consciousness and clonic movements are sometimes excluded in TND. However, the differentiation between TND and symptomatic epilepsy may be based solely on clinical features assessed by clinicians. This study included clonic movements and transient disturbances of consciousness are also included as TND [7]. TND occurred preoperatively, and often prompted patients to seek medical attention. Reports from patients or their relatives were considered as part of the clinical history; however, the final determination of TND was based on clinical features assessed by clinicians.

**Fig. 2:**
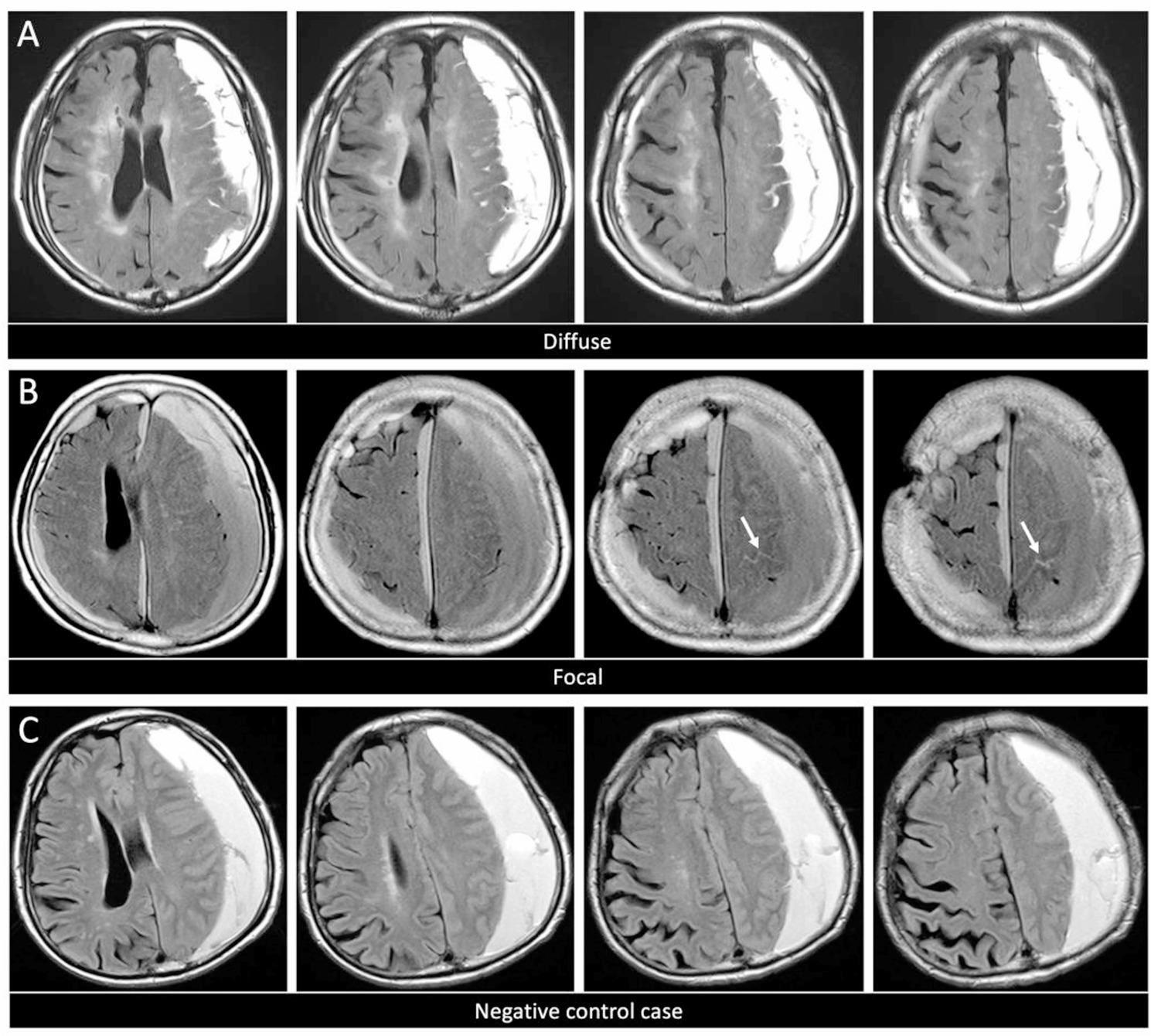
Sulcal hyperintensity on FLAIR in a patient with chronic subdural hematoma. **A** Diffuse sulcal hyperintensity (involving two lobes). **B** Focal sulcal hyperintensity (confined to a single lobe, white arrow). **C** Negative control case. Abbreviations: FLAIR; fluid attenuated inversion recovery

Hematoma location on CT was classified as left, right, or bilateral. Hematoma thickness and midline shift (mm) were measured [14]. The maximum hematoma thickness was measured on the axial slice where the hematoma length was greatest, as the maximum width perpendicular to the long axis. Midline shift was defined as the maximum displacement at the level of the foramen of Monro. For bilateral hematoma, the hematoma thickness was defined as the sum of the thicknesses of the left and right hematomas. Hematomas were classified into low-density, isodense, high-density, laminar, mixed-density, Neveau, and trabecular on CT [11, 15]. We focused on the presence or absence of SHI on FLAIR MRI. SHI on FLAIR imaging was defined as marked hyperintensity in the cerebrospinal fluid (CSF) space of ≥ 1 cortical sulci [16]. Furthermore, SHI on FLAIR was defined as marked hyperintensity along cerebral sulci adjacent to the hematoma, categorized as “diffuse” (involving two lobes) or “focal” (confined to a single lobe) (Fig. 2A–B) [6, 16]. A negative control case was shown in Fig. 2C.

Patient clinical and treatment data were extracted from the electronic medical records.

SHI was independently assessed by two neurosurgeons with ≥ 10 years of experience in MRI evaluation. Positive cases and those for which a consensus was reached were included; disagreement between the two neurosurgeons were resolved by a third neurosurgeon.

### Statistical analysis

Binary variables were represented as frequencies and percentages, whereas continuous variables were represented as medians with interquartile ranges. Intergroup differences were assessed using Fisher’s exact and Mann–Whitney U test for categorical and continuous variables, respectively. To identify factors associated with TND, univariate and multivariable logistic regression analyses were used to estimate odds ratios (OR) with 95% confidence intervals (CI). Variables with a *p* < 0.1 in the univariate analyses were entered into the multivariable logistic regression model to identify independent predictors. Sensitivity analyses were performed by including previously reported factors associated with TND in the multivariable model. Inter-rater agreement between the two neurosurgeons was measured using Cohen’s kappa. Statistical significance was defined as *p* < 0.05. The JMP Student Edition (SAS Institute) was used for statistical analysis.

## Results

### Patient demographics and preoperative characteristics

This study included 127 patients (median age; 82 [74–86] years; 86 male) between January 1, 2017 and March 31, 2025 (Fig. 1). Comparison of the clinical characteristics of the groups with and without diffuse SHI is shown in Table 1. Diffuse SHI was observed in 22 (17.32%) patients with CSDH. The inter-rater reliability (κ) for diffuse SHI identification was 0.76. Of the 22 patients with diffuse SHI, nine (40.91%) presented with TND, yielding a positive predictive value (PPV) of 40.9%. The sensitivity, specificity, and negative predictive value (NPV) of diffuse SHI for predicting TND were 69.2%, 88.6%, and 96.2%, respectively. The positive likelihood ratio (LR+) and negative likelihood ratio (LR−) were 6.1 and 0.35, respectively. The TND in the diffuse SHI group was higher than that in the non-diffuse SHI group (40.91% vs. 3.81%). The midline shift was larger in the non-diffuse SHI group than in the diffuse SHI group (5.2 [3.68, 8.33] mm vs. 7.4 [4.4, 10.25] mm). The incidence of low-density hematoma was higher in the diffuse SHI group than in the non-diffuse SHI group (36.36% vs. 6.67%).

**Table 1:** Baseline clinical characteristics and laboratory parameters of patients with and without diffuse sulcal hyperintensity

Characteristics	Diffuse sulcal hyperintensity		*P*-values
	Yes (*n* = 22)	No (*n* = 105)	
Age, years, median [IQR]	81 [72; 85]	82 [74; 86]	0.42
Sex, male (%)	17 (77.27)	69 (65.71)	0.33
Comorbidity			
Stroke (%)	2 (9.09)	11 (10.48)	1.00
Epilepsy (%)	0 (0)	2 (1.90)	1.00
Blood disease (%)	0 (0)	1 (0.95)	1.00
Liver disease (%)	2 (9.09)	0 (0)	0.03
Chronic kidney disease (%)	6 (27.27)	16 (15.24)	0.21
Malignant tumor (%)	2 (9.09)	12 (11.43)	1.00
Antithrombotic agent (%)	2 (9.09)	30 (28.57)	0.06
Symptoms			
Transient neurological dysfunction (%)	9 (40.91)	4 (3.81)	< 0.01
Cognitive dysfunction (%)	2 (9.09)	15 (14.29)	0.74
Headache (%)	3 (13.64)	9 (8.57)	0.44
Hemiplegia (%)	11 (50.00)	46 (43.81)	0.64
disturbance of consciousness (%)	2 (9.09)	3 (2.86)	0.21
CSDH side			
Right (%)	7 (31.82)	20 (19.05)	0.25
Left (%)	7 (31.82)	55 (52.38)	0.10
Both (%)	8 (36.36)	30 (28.57)	0.46
Maximal hematoma thickness, mm, median [IQR]	23 [18.85; 30.13]	26.2 [24; 30.7]	0.052
Midline shift, mm, median [IQR]	5.2 [3.68; 8.33]	7.4 [4.4; 10.25]	0.048
Characteristics of hematoma			
Low (%)	8 (36.36)	7 (6.67)	< 0.01
Iso (%)	2 (9.09)	13 (12.38)	1.00
High (%)	1 (4.55)	14 (13.33)	0.47
Laminar (%)	0 (0)	11 (10.48)	0.21
Mixed (%)	4 (18.18)	17 (16.19)	0.76
Neveau (%)	2 (9.09)	14 (13.33)	0.74
Trabecular (%)	3 (13.64)	20 (19.05)	0.76
mRS score, median [IQR]			
Pretreatment	1 [0; 2]	1 [0; 2]	0.97
At discharge	1 [0; 3]	1 [0; 2]	0.62
Recurrence (%)	2 (9.09)	17 (16.19)	0.52

Data are expressed as the number (%) of patients unless otherwise indicated

*Abbreviations*: *CSDH *Chronic subdural hematoma, *IQR *Interquartile, *mRS *modified Rankin Scale

### Assessment of the association between transient neurological dysfunction and diffuse sulcal hyperintensity

Univariate logistic regression analysis revealed associations between sex (unadjusted OR; 6.49, 95% CI; 1.21–120.26, *p* = 0.03) and diffuse SHI (unadjusted OR; 17.48, 95% CI; 4.98–72.48, *p* < 0.01) with TND (Table 2). In the multivariable model adjusting for sex, diffuse SHI remained independently associated with TND (adjusted OR; 17.07, 95% CI; 4.71–73.23, *p* < 0.01). The association between male sex and TND was attenuated and no longer statistically significant after adjustment (adjusted OR: 6.16, 95% CI: 0.99-120.78, *p* = 0.051).

**Table 2:** Univariate and multivariable logistic regression analysis of factors associated with transient neurological dysfunction

Variables	Unadjusted OR (95% CI)	*P*-value	Adjusted OR (95% CI)	*P*-value
Age	0.99 (0.93–1.06)	0.78		
Sex (Ref = female)	6.49 (1.21–120.26)	0.03	6.16 (0.99–120.78)	0.051
Stroke	1.70 (0.24–7.48)	0.54		
Epilepsy	9.42 (0.36–248.49)	0.15		
Blood disease	NA	NA		
Liver disease	NA	NA		
Chronic kidney disease	2.37 (0.59–8.17)	0.21		
Malignant tumor	0.65 (0.03–3.72)	0.67		
Antithrombotic agent	0.88 (0.19–3.11)	0.85		
Diffuse sulcal hyperintensity	17.48 (4.98–72.48)	< 0.01	17.07 (4.71–73.23)	< 0.01
Focal sulcal hyperintensity	0.44 (0.02–2.48)	0.40		
Right	1.76 (0.45–5.94)	0.40		
Left	0.63 (0.18–1.99)	0.43		
Both	1.05 (0.27–3.45)	0.94		
Maximal hematoma thickness	0.98 (0.90–1.05)	0.58		
Midline shift	0.95 (0.80–1.11)	0.56		
Low	2.55 (0.52–9.79)	0.23		
Iso	1.41 (0.20–6.06)	0.68		
High	0.60 (0.03–3.40)	0.61		
Laminar	0.87 (0.05–5.16)	0.89		
Mixed	2.54 (0.63–8.79)	0.18		
Neveau	0.55 (0.03–3.12)	0.55		
Trabecular	NA	NA		
Pretreatment mRS	1.14 (0.72–1.74)	0.57		
At discharge mRS	1.33 (0.90–1.98)	0.15		
Recurrence	1.04 (0.15–4.32)	0.96		

*Abbreviations*: *OR *Odds ratio, *CI *Confidence interval, *Ref *Reference, *mRS *modified Rankin Scale, *NA *Not assessed

Sensitivity analysis was performed using mixed density hematoma, sex (men), and left side hematoma as TND-associated factors [8, 11]. This revealed that diffuse SHI was an independent factor (Supplemental Table S1-3).

## Discussion

This study demonstrated a significant correlation between TND and diffuse SHI in patients with CSDH. The association between male sex and TND was attenuated and no longer statistically significant after adjustment. In a previous report, 82 of 113 CSDH patients (73%) with TND were male. The proportion of males was similarly high in the non-TND group (876 of 1,194 patients, 74%), with no statistically significant difference between groups. These findings suggest that the high proportion of males in the TND group likely reflects the epidemiological characteristic that CSDH itself is more common in men [7]. The association between TND and SHI has been previously documented in case reports and small-scale cohort studies in Table 3. A case series reported three cases of CSDH with SHI that emerged during TND, suggesting that SHI is a crucial predictor of TND [17]. Recent literature reported that diffuse SHI was observed in 12 out of 13 cases (92.3%) with TND, showing a significant association if compared with cases without SHI or those with only focal SHI [6]. Additionally, several reports have indicated a relationship between SHI and seizures, providing insights into elucidating the mechanisms of TND. A case report showed that middle meningeal artery embolization was performed in patients with CSDH with convulsions, and the disappearance of SHI was confirmed [9]. Additionally, a study reported that the cortical regions showing SHI displayed concordance between ictal EEG and perfusion areas identified by arterial spin labeling [18]. Furthermore, another report demonstrated a transient decrease in the cortical benzodiazepine receptor binding potential (BRBP) on the side corresponding to SHI on 123I-iomazenil SPECT, confirming concordance with the epileptic focus [10]. A single-center observational study revealed that SHI was significantly higher in the group of patients who experienced seizures perioperatively than in the non-seizures (seizure group; 45.0%, non-seizure group; 12.5%, p=0.009) [11]. Seizures may arise from several potential mechanisms, such as localized decreases in cerebral blood flow owing to compression from a hematoma, injury to the cerebral cortex, and gliosis associated with the development of a hematoma capsule. As TND is a symptom-based concept, it may include various pathophysiological conditions, such as specific types of epileptic activity and cortical spreading depression [6].

**Table 3:** Summary of reported studies on TND and SHI

Author and Year	Study design	Patient population	Definition of SHI	Main findings
Takahashi et al., 2025 [17]	Case series	3 SDH patients with TND, no surgery	NI	SHI in SDH may be an important finding predictive of TND.
Kato et al., 2025 [6]	Retrospective	72 CSDH patients undergoing burr-hole surgery	SHI on FLAIR was defined as marked hyperintensity along ipsilateral cerebral sulci and categorized SHI as diffuse ( ≧ 2 lobes) or focal (single lobe)	Combination of diffuse SHI and W/N on MRI strongly correlates with TNDs in patients with CSDH
Hasegawa et al., 2021 [9]	Case report	A CSDH patient with seizures undergoing middle meningeal embolization	NI	Middle meningeal artery embolization is effective for CSDH with SHI.
Mugita et al., 2025 [18]	Retrospective	34 CSDH patients undergoing burr-hole surgery	NI	SHI is a nonspecific finding caused by alterations in regional hemodynamics, including increased S-ATA levels with prolonged ATT due to mass effect of CSDH.
Oshida et al., 2019 [10]	Case report	A CSDH patient with seizures undergoing burr-hole surgery	NI	SHI corresponded to the epileptic focus.
Neshige et al., 2014 [11]	Retrospective	20 CSDH patients with seizures undergoing burr-hole surgery	NI	SHI was significantly more frequent in the seizure group than in the non-seizure group.

*Abbreviations*: *ATT *Arterial transit time, *CSDH *Chronic subdural hematoma, *NI *No information, *S-ATA *Sulcal arterial transit artifact, *SDH *Subdural hematoma, *TND *Transient neurological deficit, *W/N *Web/net appearance on T2^༊^-weighted images

Two main factors were assumed to be involved in the SHI mechanism. Firstly, local hemodynamic changes may have contributed to this process. Venous congestion, combined with the mass effect of CSDH, increases abnormal ratio of blood accumulation in the cerebrospinal fluid in the cerebral sulci. Consequently, suppression of the cerebrospinal fluid signal on FLAIR images is inadequate, resulting in high signal intensity. Furthermore, observations, such as increased sulcal arterial transit artifacts on the side of the CSDH, which indicate a prolonged arterial transit time, have been documented. These findings suggest that alterations in local hemodynamics due to mass effects contribute to SHI development [18]. Secondly, SHI is observed in inflammatory conditions, such as meningitis, carcinomatous meningitis, and subarachnoid hemorrhage. Stimulation of the meninges through protein leakage into the subarachnoid space may be important in SHI [10]. The hematoma membrane in CSDH is rich in neovascularization and highly permeable. Particularly, in hematomas with high fibrinolytic activity (mixed-type hematomas), substances containing fibrin degradation products may permeate the hematoma membrane and affect the brain parenchyma and meninges, potentially contributing to SHI [11]. Conversely, only approximately 31% of the patients with CSDH and TND who showed SHI had mixed-type hematomas, and no significant association was identified between TND and mixed-type hematomas. Previous reports have suggested an association between seizures and mixed-type hematomas, however, in this study, seizures were included in the definition of TND. Therefore, it is necessary to consider that previous reports and the underlying assumptions differ, and further examination is required to interpret this discrepancy.

This study classified SHI into diffuse and focal types. In this study, diffuse SHI was significantly associated with TND, whereas focal SHI was not significantly associated with TND. Focal SHI is likely to reflect localized hemodynamic changes caused by local mass effect or compression, whereas diffuse SHI reflects widespread blood flow abnormalities (particularly congestion or extensive inflammation) associated with vascular diseases, indicating differences in their pathophysiology [16]. Pathological examinations revealed that CSDH presenting with diffuse SHI and Web/Net Appearance on T2* showed inflammatory cell infiltration, abundant neovascularization, and proliferation of fibroblasts in the outer membrane [6]. Previously, although sampling of the inner membrane tissue was not performed because of concerns over complications, CSDH membranes were characterized by the presence of numerous thin-walled, permeable capillaries that lack basal nuclei, smooth muscle cells, and pericytes [19, 20]. This fragile structure is considered a possible contributor to the spread of inflammation in the cerebral cortex. Such diffuse inflammation of the cerebral cortex may be involved in epileptogenesis. Diffuse SHI may reflect a condition in which recurrent bleeding within an organized hematoma triggers an inflammatory response, and this inflammation spreads to the adjacent brain surface through the inner membrane, potentially disrupting the blood-brain barrier [6]. However, although a strong association has been suggested between the presence of SHI and TND, a causal relationship remains debated. Mugita et al. indicated that SHI is a nonspecific finding observed preoperatively in approximately one-third of the patients with CSDH and proposed that the underlying cause was delays in arterial transit time; however, this is not directly involved in the onset of seizures [18]. Moreover, none of the two patients that developed seizures showed SHI preoperatively, and in one of these patients, a new SHI appeared after the seizure on postoperative day 7. Previous reports suggest that in some cases, MRI was performed after the occurrence of complications, such as seizures, and SHI was retrospectively detected. This study found that TND was absent in 13 of the 22 instances (59%) of diffuse SHI, and no new evidence was found to support a causal link between the two. This study provides evidence supporting the connection between the pathophysiological mechanisms involved in TND and pathogenesis and pathological features of SHI, highlighting their similarities. Regarding the diagnostic performance of diffuse SHI, the NPV was high (96.2%), indicating that patients without diffuse SHI rarely developed TND in this cohort. Furthermore, the likelihood ratios (LR+ 6.1 and LR− 0.35) suggest that diffuse SHI has a moderate ability to increase the probability of TND when present while substantially decreasing its likelihood when absent. These findings suggest that diffuse SHI may be particularly useful as a rule-out imaging marker. Diffuse SHI may serve as a potential biomarker for TND in patients with CSDH, providing useful information that can complement existing diagnostic methods; however, further investigation is necessary to clarify its causal role.

This study had some limitations. First, this was a retrospective and single-center study. There were limitations to accurately evaluating the prevalence of SHI in patients with CSDH who did not undergo MRI. Furthermore, the number of patients with diffuse SHI (n = 22) and the number of TND events (n = 13) were relatively small, which may increase the risk of overfitting in the statistical model, especially with a binary outcome. Therefore, the findings are preliminary and require validation in a larger, prospective cohort. Second, although the focus was on the relationship between MRI findings and TND, the underlying pathophysiology remains unknown. For a more comprehensive understanding of the pathophysiology of TND, assessments, such as MRI, perfusion CT, and electroencephalography, must be incorporated [8]. However, because TND is a transient symptom and there are cases in which patients seek medical attention after their symptoms improve, it is difficult to capture the sudden onset of this condition through imaging findings. However, SHI reflects findings indicative of inflammation and represents an acute course. To overcome these challenges, research designs that directly assess underlying electrophysiological mechanisms are required. Third, although the association between diffuse SHI and TND was statistically significant, the corresponding odds ratios showed wide confidence intervals. This reflects statistical imprecision in the effect estimates, which is likely due to the limited sample size, particularly the small number of TND cases. Therefore, while the true magnitude of the relationship between diffuse SHI and TND should be interpreted with caution and confirmed in larger cohorts.

## Conclusions

This study demonstrates a significant correlation between preoperative TND and the presence of diffuse SHI in patients with CSDH. These findings may serve as a potential biomarker for TND in patients with CSDH, providing useful information that can complement existing diagnostic methods.

## Supplementary Information

Supplementary Material 1.

## Data Availability

The patient data used in this study are not publicly available due to the presence of personal andsensitive patient information. The datasets used and/or analyzed during the current study are availablefrom the corresponding author on reasonable request with appropriate institutional approvals.

## Abbreviations

CI: Confidence intervals
CSDH: Chronic subdural hematoma
CSF: Cerebrospinal fluid
CT: Computed tomography
FLAIR: Fluid attenuated inversion recovery
MRI: Magnetic resonance imaging
mRS: Modified Rankin Scale
NPV: Negative predictive value
OR: Odds ratios
PPV: Positive predictive value
SHI: Sulcal hyperintensity
TND: Transient neurological deficits
