# Supplementary material for: Diffuse sulcal hyperintensity on MRI as a radiological correlate of transient neurological deficits in chronic subdural hematoma
Source: BMC Neurol. 2026 Apr 23;26:370. doi: 10.1186/s12883-026-04913-6 (PMC13237949; doi:10.1186/s12883-026-04913-6)
Supplement: Supplementary file 1 — Supplementary Material 1. [file 12883_2026_4913_MOESM1_ESM.docx]

**Supplementary Materials**

**Table S1** Sensitivity analysis for the relationship between diffuse sulcal hyperintensity and transient neurological dysfunction (multiple imputation for missing values).

| **Variable** | **Adjusted OR (95% CI)** | ***p****-***value** |
| --- | --- | --- |
| Mixed density hematoma | 0.05 (0.01–0.19) | 0.15 |
| Diffuse sulcal hyperintensity | 18.75 (5.17–82.46) | <0.01 |

Abbreviations: OR, odds ratio; CI, confidence interval

**Table S2** Sensitivity analysis for the relationship between diffuse sulcal hyperintensity and transient neurological dysfunction (multiple imputation for missing values).

| **Variable** | **Adjusted OR (95% CI)** | ***p****-***value** |
| --- | --- | --- |
| Male | 6.16 (0.99–120.78) | 0.051 |
| Diffuse sulcal hyperintensity | 17.07 (4.71–73.23) | <0.01 |

Abbreviations: OR, odds ratio; CI, confidence interval

**Table S3** Sensitivity analysis for the relationship between diffuse sulcal hyperintensity and transient neurological dysfunction (multiple imputation for missing values).

| **Variable** | **Adjusted OR (95% CI)** | ***p****-***value** |
| --- | --- | --- |
| Left side hematoma | 1.02 (0.26–4.03) | 0.98 |
| Diffuse sulcal hyperintensity | 17.55 (4.87–75.29) | <0.01 |

Abbreviations: OR, odds ratio; CI, confidence interval
